# Identification of Aberrantly-Expressed Long Non-Coding RNAs in Osteoblastic Cells from Osteoporotic Patients

**DOI:** 10.3390/biomedicines8030065

**Published:** 2020-03-19

**Authors:** Federica Centofanti, Massimo Santoro, Mario Marini, Virginia Veronica Visconti, Anna Maria Rinaldi, Monica Celi, Giovanna D’Arcangelo, Giuseppe Novelli, Augusto Orlandi, Virginia Tancredi, Umberto Tarantino, Annalisa Botta

**Affiliations:** 1 Department of Biomedicine and Prevention, Anatomic Pathology Section, University of Rome “Tor Vergata”, 00133 Rome, Italy; federica.centofanti@uniroma2.it (F.C.); orlandi@uniroma2.it (A.O.); 2 IRCCS Don Gnocchi Foundation, 20121 Milan, Italy; masantoro@dongnocchi.it; 3 Department of Systems Medicine, University of Rome “Tor Vergata”, 00133 Rome, Italy; mario.marini@uniroma2.it (M.M.); annamaria.rinaldi@uniroma2.it (A.M.R.); giovanna.darcangelo@uniroma2.it (G.D.); tancredi@uniroma2.it (V.T.); 4 Center of Space Biomedicine, University of Rome Tor Vergata, 00133 Rome, Italy; 5 Department of Biomedicine and Prevention, Medical Genetics Section, University of Rome “Tor Vergata”, 00133 Rome, Italy; virginia.veronica.visconti@uniroma2.it (V.V.V.); novelli@med.uniroma2.it (G.N.); 6 Department of Clinical Sciences and Translational Medicine, University of Rome "Tor Vergata”, 00133 Rome, Italy; monica.celi@uniroma2.it; 7 IRCCS Neuromed, Unit of Medical Genetics, Via Atinense 18, 86077 Pozzilli, Italy; 8 Department of Orthopedics and Traumatology, PTV Foundation, 00133 Rome, Italy

**Keywords:** osteoporosis, epigenetics, long non-coding RNAs, inflammation, bone homeostasis, osteoblast

## Abstract

Osteoporosis (OP) is a multifactorial disease influenced by genetic, epigenetic, and environmental factors. One of the main causes of the bone homeostasis alteration is inflammation resulting in excessive bone resorption. Long non-coding RNAs (lncRNAs), have a crucial role in regulating many important biological processes in bone, including inflammation. We designed our study to identify lncRNAs misregulated in osteoblast primary cultures derived from OP patients (*n* = 4), and controls (CTRs, *n* = 4) with the aim of predicting possible RNA and/or protein targets implicated in this multifactorial disease. We focused on 84 lncRNAs regulating the expression of pro-inflammatory and anti-inflammatory genes and miRNAs. In silico analysis was utilized to predict the interaction of lncRNAs with miRNAs, mRNAs, and proteins targets. Six lncRNAs were significantly down-regulated in OP patients compared to controls: *CEP83-AS1*, *RP11-84C13.1*, *CTC-487M23.5*, *GAS5*, *NCBP2-AS2*, and *SDCBP2-AS1*. Bioinformatic analyses identified HDCA2, PTX3, and FGF2 proteins as downstream targets of *CTC-487M23.5, GAS5*, and *RP11-84C13.1* lncRNAs mediated by the interaction with miRNAs implicated in OP pathogenesis, including *miR-21-5*p. Altogether, these data open a new regulatory mechanism of gene expression in bone homeostasis and could direct the development of future therapeutic approaches.

## 1. Introduction

Osteoporosis (OP) is characterized by an imbalance in the regulation of bone remodeling between osteoblastic bone formation and osteoclastic bone resorption [1]. Currently OP is viewed as a heterogeneous condition which can occur in any age of life and its etiology is attributed to various endocrine, metabolic, and mechanical factors. Among them, hormonal deficiency plays a fundamental role. Estrogen has a protective effect in bone, due to a direct action on osteoclasts (increasing apoptosis and reducing the receptor activator of nuclear factor-κB ligand (RANKL)-dependent osteoclast formation) and osteoblasts (increasing osteoblast survival and collagens type I production) [2]. Bone homeostasis is important to maintain the coordinated activity between bone-forming osteoblasts and bone-resorbing osteoclasts. Emerging evidence suggests that bone homeostasis imbalance is caused by estrogen deficiency resulting in increased inflammation that results in excessive bone resorption. Osteoporosis is therefore described as an inflammatory disease [2]. In such inflamed situations, pro-inflammatory cytokines such as TNF-α, IL-1β, and IL-6 stimulate osteoclastogenesis directly. This leads to the recruitment of osteoclast precursor cells into the bone-erosive site and their subsequent differentiation into mature osteoclasts [3].

Numerous genes have been associated with osteoporosis risk and among candidate genes, the *vitamin D receptor* (*VDR*) gene, encoding a nuclear hormone receptor, was the first to be proposed as a major susceptibility locus [4]. Recently, genome-wide association studies (GWASs) have identified several osteoporosis-related genes, such as *catenin* (*cadherin-associated protein*) *beta 1* (*CTNNB1*), *sclerostin* (*SOST*), *low-density lipoprotein receptor-related protein 4* (*LRP4*), *LRP5*, *wingless-type MMTV integration site family, member 4* (*WNT4*), *WNT5B*, and *AXIN1* [5]. However, genetic factors alone are not sufficient to explain the pathogenesis of osteoporosis. Epigenetic factors represent a link between individual genetic aspects and environmental influences, and they are involved in bone biology and osteoporosis [6]. These mechanisms include post-translational histone modifications, ncRNA-mediated post-transcriptional regulation, and DNA methylation [7]. Several studies showed the importance of miRNAs in controlling bone homeostasis and metabolism. In particular, in a study performed by Weilner et al., authors have found 10 circulating miRNAs deregulated in patients with recent osteoporotic fractures. In total six miRNAs, *miR-10a-5p*, *miR-10b-5p*, *miR-133b*, *miR-22-3p*, *miR-328-3p*, and *let-7g-5p* exhibited significantly different serum levels in response to fracture [8]. Another two studies have profiled the expression of miRNAs in peripheral blood mononuclear cells (PBMCs) from postmenopausal women with low and high bone mineral density (BMD), and they found that *miR-503* was markedly reduced and *miR-133a* significantly increased in OP patients [7]. More recently, much interest has focused on another class of ncRNAs, long non-coding RNAs (lncRNAs) belonging to a novel heterogeneous class of non-protein-coding transcripts with a length of more than 200 nt [1] and with many different functions. LncRNAs can act as decoys by binding to RNA or proteins, they can inhibit or promote transcription through histone and chromatin alteration, alter splicing profiles, or mask miRNA binding sites [9]. LncRNAs have crucial roles in regulating many important biological processes such as cell proliferation, differentiation, migration, and development thus contributing to molecular pathogenesis of different human diseases [1]. To date, studies on the role of lncRNAs in bone biology have been limited. In one study, it was proposed that *DANCR* (*RNA- differentiation antagonizing nonprotein coding RNA*) lncRNA regulates expression of the osteogenic regulator Runx2 by recruiting the Polycomb repressive complex component EZH2 to the *Runx2* promoter, thereby resulting in suppression of osteogenesis [10]. Runx2 is a member of the RUNX family of transcription factors and encodes for a nuclear protein with a Runt DNA-binding domain and is essential for osteoblast development and bone formation. Runx2 drives pluripotent mesenchymal cells to the osteoblast lineage and increases their maturation and function by regulating the expression of several osteoblast-related extracellular matrix proteins, in particular osteocalcin (OC) [11]. Otto et al. demonstrated that *Runx2*-deficient mice present a cartilaginous skeleton associated with the absence of osteoblasts, highlighting the major role of *Runx2* in bone patterning and endochondral and intramembranous ossification [12]. Additionally, a set of lncRNAs microarray expression data was also generated from a mouse mesenchymal stem cells (MSC) line that was stimulated with BMP2. This study identified 116 lncRNAs that were differentially expressed but no functional characterization was performed to test the requirements for these lncRNAs in osteogenesis [13]. 

Primary cultures are important tools to provide valuable information about the processes of skeletal development, bone formation, and bone resorption [14]. In this study, we analysed the expression profile of 84 lncRNAs validated or predicted to regulate the expression of genes for acute-phase response, autoimmunity, humoral immune response, inflammatory responses, and innate and adaptive immunity in cultured primary osteoblast cells from OP patients (*n* = 4) and control (CTR) individuals (*n* = 4) in order to find a possible regulatory mechanism controlling changes in gene expression in bone homeostasis.

## 2. Experimental Section

### 2.1. Subjects

We enrolled eight individuals who underwent hip surgery in the Orthopedic and Traumatology Department of Policlinico Tor Vergata Hospital. Specifically, we enrolled four consecutive patients (three females and one male) (OP group) who underwent hip arthroplasty for medial hip fractures for low-energy trauma (73.00 ± 3.20 years), and four individuals (44.23 ± 2.77 years) who underwent hip arthroplasty for high-energy hip fractures (two females and two males) (CTR, group) (Table 1). Exclusion criteria were history of cancer, myopathies, or other neuromuscular diseases or chronic administration of corticosteroid for autoimmune diseases (> 1 month), diabetes, alcohol abuse and HBV, HCV, or HIV infections.

All experiments described in the present study were approved by the ethics committee of Policlinico Tor Vergata (approval reference number #85/12; June, 2017). All experimental procedures were carried out according to The Code of Ethics of the World Medical Association (Declaration of Helsinki). Informed consent was obtained from all patients prior to surgery. Specimens were handled and carried out in accordance with the approved guidelines.

### 2.2. Human Osteoblast Primary Cell Cultures

**Figure 1:**
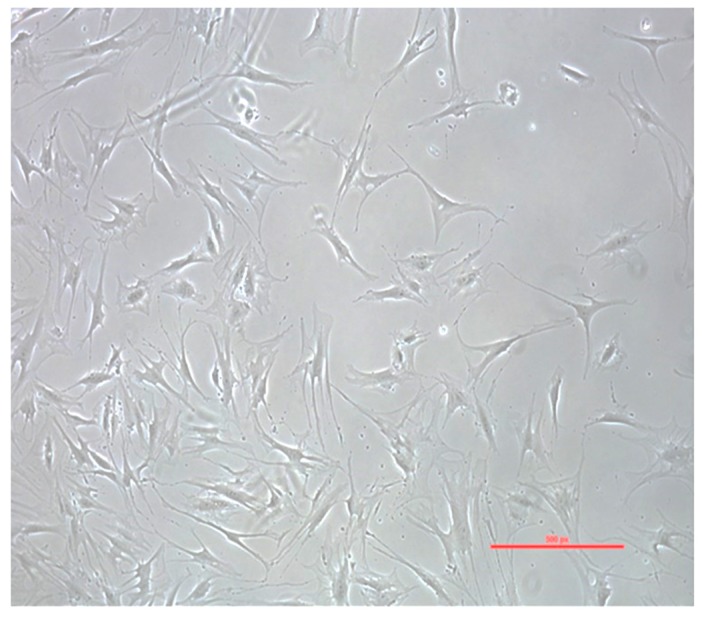
Primary human osteoblast cultures were observed under phase-contrast microscope at 10× field. Scale bar: 500 μm.

Primary cultures of osteoblasts were isolated from the cancellous bone of healthy patients with high-energy femoral fracture and patient affected by osteoporosis. The samples were dissected and treated to obtain a homogeneous population of osteoblasts. Briefly, after dissection, trabecular bone fragments were repeatedly washed in PBS. Then bone fragments were briefly incubated at 37 °C with 1 mg/mL trypsin from porcine pancreas ≥ 60 U/mg (SERVA Electrophoresis GmbH, Heidelberg, Germany) diluted in DPBS. After washing, bone fragments were subjected to repeated digestions with 2.5 mg/mL Collagenase NB 4G Proved grade ≥ 0.18 U/mg (SERVA Electrophoresis GmbH) diluted in DPBS with calcium and magnesium. Supernatant were collected and centrifuged at 310 RCF (Eppendorf, 5804 R) for 5 min. Cell pellets were resuspended in DMEM/F12 (Biowest, Nuaillé, FR) supplemented with 15% FBS, 50 μg/mL gentamicin, and 0.08% Fungizone, penicillin streptomycin (Sigma Chemical Co., St. Louis, MO, USA), and amphotericin B (Biowest), seeded into a 24-well plate and incubated at 37 °C, 5% CO_2_ until reaching confluence (about 4 weeks). Medium was changed twice a week (Figure 1) [15,16,17]. To assess the quality of each cell purification a fraction of the purified cells was inspected using immunochemistry analysis and morphological inspection. Osteoblast primary cells were observed to be homogeneous and appropriate for osteoblasts and expressing BMP2, RUNX2, and RANK-L. Cells were analyzed for lncRNAs expression at third passage. 

### 2.3. LncRNA Analysis

Total RNA was extracted from osteoblast primary cultures derived by OP (*n* = 4) and CTR (*n* = 4), as described [18]. First strand cDNA was synthesized using RT^2^PreAMP cDNA synthesis kit (QIAGEN, Germany) and pre-amplified with RT^2^lncRNA PreAMP primer mix (QIAGEN, Hilden, Germany) that contained a specific set of primers to target genes of the human RT^2^lncRNA inflammatory response and the autoimmunity PCR array (QIAGEN, Hilden, Germany). qRT-PCR was performed using RT^2^ SYBR^®^ Green qPCR MasterMix (QIAGEN, Hilden, Germany) and RT^2^lncRNA PCR array human inflammatory response and autoimmunity (QIAGEN, Hilden, Germany) which contains pre-dispensed, laboratory verified, gene-specific primer pairs). The experiment was designed to analyze each sample on a single 96-well plate. The reaction was performed by ABI 7500 Fast Real-Time PCR System (Applied Biosystems, Foster City, CA, USA) with a holding stage at 95 °C for 10 min followed by 40 cycles of each PCR step: 95 °C for 15 s and 60 °C for 1 min. For data analysis, the Ct values were exported to an Excel file and uploaded into the RT^2^ PCR Array Data Analysis Web Portal at https://www.qiagen.com/dataanalysiscenter. Ct values were normalized based on automatic selection of housekeeping from the full panel on the PCR array. This method automatically selected two optimal reference genes *RP11-325K4.3* (ENST00000565861) and *RP11-96D1.10* (ENST00000571975) with the most stable expression for the analysis from the full plate on the PCR array. Using the Data Analysis Web Portal, we calculated fold change/regulation with *ΔΔC_t_* method, in which *ΔC_t_* is calculated between gene of interest and an average of reference genes, followed by *ΔΔC_t_* calculations (*ΔC_t_* (patient) − *ΔC_t_* (control)). Fold change was then calculated using the 2^−ΔΔCt^ formula. The *p*-values were calculated based on a Student’s t test of the three replicate 2^(−ΔCt)^ values for each gene in the control and OP groups. 

### 2.4. Prediction Methods for lncRNA–RNA Interactions

In silico prediction of lncRNA–mRNA interactions was performed using the database containing the lncRNA–mRNA and lncRNA–lncRNA interactions for 23,898 lncRNAs and 20,185 mRNAs (available at http://rtools.cbrc.jp/cgi-bin/RNARNA/index.pl). In this database, each possible pair of RNAs are ranked according to two interaction energies calculated from the local interaction energies between the two RNA sequences: MinEnergy (adequate for short RNA sequences or for cases in which the strongest local interaction is dominant) and SumEnergy (adequate for long RNA sequences such as lncRNAs and mRNAs and for cases in which several strong interactions exist). The predicted lncRNA–mRNA interactions including these two types of interacting energies were used for the analysis [19].

### 2.5. Prediction Methods for lncRNA–RNA Binding Protein (RBP) Interactions

RBP and lncRNA interactions were predicted using the NPInter v3.0 (https://www.bioinfo.org/NPInter/index.htm), a database which contains experimentally-verified interaction between ncRNA (excluding tRNA and rRNA) and other biomolecules (proteins, mRNA, miRNA, and genomic DNAs) [20].

### 2.6. Prediction Methods for lncRNA–miRNA Interactions

In silico prediction of lncRNA–miRNA interactions was performed using DIANA-LncBase v2 [21] (www.microrna.gr/LncBase), a database of experimentally-supported and in silico-predicted miRNA recognition elements (MREs) on lncRNAs, identified with the DIANA-microT algorithm. For assessment of miRNA regulatory roles in bone homeostasis and osteoporosis pathways and the identification of miRNA-target genes implicated in these pathways, we performed a functional characterization using DIANA-miRPath v3.0 [22] (http://www.microrna.gr/miRPathv3) and DIANA-TarBase v8.0 [23] (http://carolina.imis.athena-innovation.gr/diana_tools/web/index.php?r=tarbasev8%2Findex), respectively.

## 3. Results

### 3.1. Clinical Evaluation and In Vitro Study of Human Osteoblast Primary Cell Cultures

The OP group included four patients (three females and one male) with fragility hip fracture, T-score ≤ −2.5 S.D. and Kellgren−Lawrence (K–L) score from 0 to 1; while four control individuals (two females and two males) were characterized by a T-score ≥ −1.0 S.D. and K–L score from 0 to 1 (Table 1). Osteoblasts from the cancellous bone of OP and control (CTR) individuals were isolated and cultured in vitro (Figure 1), as described in the Methods section, for lncRNAs expression analysis.

### 3.2. Expression Profile of lncRNAs

**Figure 2:**
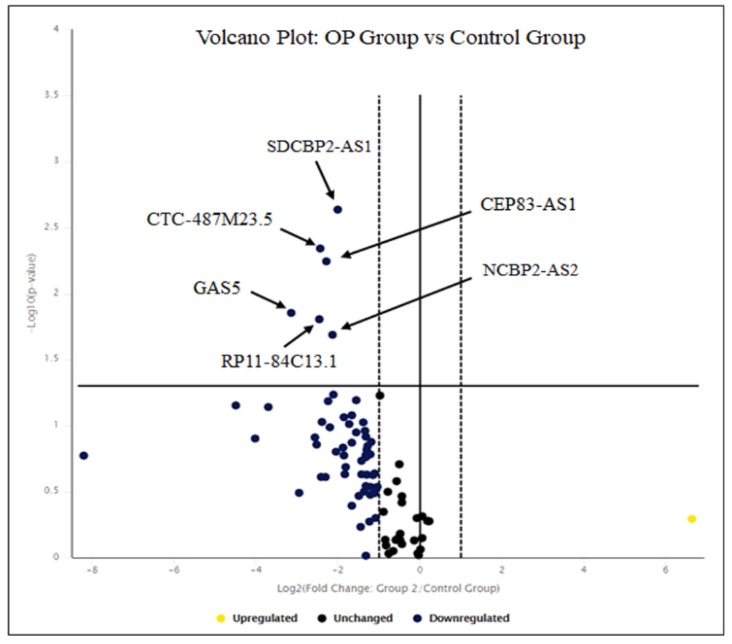
Volcano plot of LncRNAs levels in OP *vs*. CTRs. The Volcano plot shows the significant lncRNAs that we found down-regulated in osteoblast cell lines on the left (down-regulated) and above the solid vertical line (statistically significant), according to significant *p*-value and fold-regulation change. The solid vertical line represents no change in gene expression (log2 (1) = 0). The dotted lines represent a selected threshold or boundary for fold change. The default setting is 2. The solid vertical line represents a selected threshold for the *p*-value and statistical significance. The default setting is 0.05. Statistical significance versus fold-change is shown on the y- and x-axes, respectively [24].

In order to evaluate the expression levels of lncRNAs in osteoblast primary cells from OP patients vs. CTRs, we analyzed 84 lncRNAs, validated or predicted to regulate the expression of pro-inflammatory and anti-inflammatory genes and microRNAs (https://www.qiagen.com/us/shop/assay-technologies/realtime-pcr-and-rt-pcr-reagents/rt2-lncrna-pcr-arrays?catno=-rt-pcr-reagents/rt2-lncrna-pcr-arrays?catno=LAHS-004Z#geneglobe). We selected significant changes (*p* < 0.05) that were at least two-fold up- or down-regulated as compared to controls. We identified six lncRNAs that were significantly down-regulated in OP patients compared to CTRs: *CEP83 antisense RNA 1* (*CEP83-AS1* and NR_027035) fold-regulation = −8.75 (*p* = 0.014), *RP11-84C13.1* (ENST00000603357) fold regulation= −5.44 (*p* = 0.016), *CTC-487M23.5* (ENST00000602872) fold-regulation = −5.39 (*p* = 0.004), *growth arrest-specific 5* (*GAS5* and NR_002578) fold-regulation= −4.86 (*p* = 0.006), *hypothetical LOC152217* (*NCBP2-AS2* and NR_024388) fold-regulation= −4.34 (*p* = 0.02), *SDCBP2 antisense RNA 1* (*SDCBP2-AS1* and ENST00000446423) fold-regulation= −4.00 (*p* = 0.002) (Table 2 and Figure 2).

### 3.3. Bioinformatic Analysis and Target Prediction

In order to investigate a possible role of the six lncRNAs differentially expressed in primary osteoblasts from OP patients, we perform a bioinformatic analysis using different web-based target prediction tools for mRNAs, proteins, or miRNA targets.

First, we identified putative lncRNA–mRNA interactions using a database that contains all the predicted RNA−RNA interactions along with the following information: MinEnergy and SumEnergy [19] (http://rtools.cbrc.jp/cgi-bin/RNARNA/index.pl). The top five predicted RNA targets according to both MinEnergy and SumEnergy interaction are reported in Table 3. This analysis did not uncover any RNA molecule interacting with *CEP83 antisense RNA 1* (head to head) lncRNA.

We then searched for lncRNA–protein interactions with the aim of identifying proteins implicated in the pathogenic mechanism of osteoporosis. This in silico analysis was performed through the NPInter v3.0 database and disclosed only two protein targets potentially related to bone homeostasis: HNRNPC [25] interacting with *GAS5* and ADAR1 [26] interacting with *SDCBP2-AS1* lncRNA.

We also used DIANA-LncBase v2 tools to identify miRNAs targeted by the six lncRNAs deregulated in osteoblast cell lines. This analysis revealed that *CTC-487M23.5*, *GAS5*, and *RP11-84C13.1* lncRNAs potentially bind only one miRNA each: *hsa-miR-136-3p* (Pr. Score 0.451), *hsa-miR-21-5p* (Pr. Score 0.382), and *hsa-miR-576-3p* (Pr. Score 0.408), respectively (Table 4). Conversely, *NCBP2-AS2* and *SDCBP2-AS1* lncRNAs interact with multiple miRNAs, including *miR-497~195* cluster, *hsa-miR-103a-3p*, *hsa-miR-23a-3p*, *hsa-miR-24-2-5p*, and *hsa-miR-23b-3p* which have been already associated with the inhibition of osteoblastogenesis (through the downregulation of multiple BMP-responsive genes), regulation of osteoblast differentiation (by directly targeting Runx2), and suppression of osteogenesis and bone formation by targeting most of the known signaling pathways involved in osteoblast biology [27,28].

Finally, DIANA-miRPath v3.0 and DIANA-TarBase v8.0 have been queried to understand the regulatory role of miRNAs into biological processes and pathways through the identification of targeted proteins. As shown in Table 4, the downregulation of *CTC-487M23.5*, *GAS5*, *RP11-84C13.1*, *NCBP2-AS2*, and *SDCBP2-AS1* could interfere with bone homeostasis deregulating different KEEG pathway such as Hippo, Wnt and FoxO signaling related to bone formation and resorption. Unfortunately, we were not able to identify any *CEP83-AS1* protein interactors. 

## 4. Discussion

**Figure 3:**
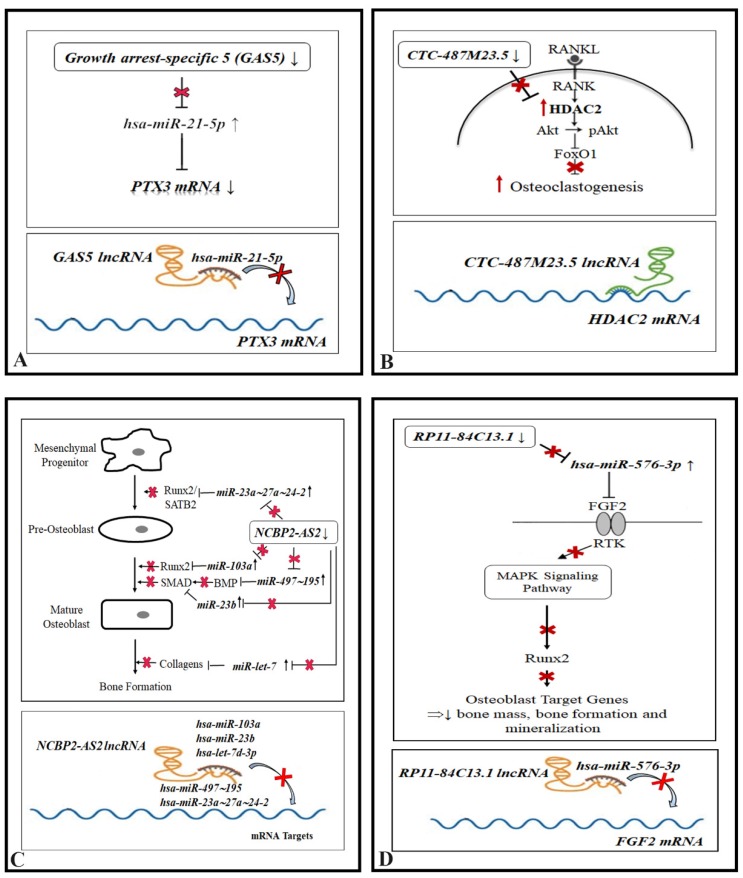
Hypothetical pathogenetic mechanisms emerging from this study. (**A**) *Growth arrest-specific 5* (*GAS5*) lncRNA masks *miR-21-5p* binding sites, (**B**) *CTC-487M23.5* lncRNA inhibits the translation of *HDAC2* mRNA, (**C**) *NCPB2-AS2* lncRNA masks miRNA binding sites implicated in osteoblast differentiation, and (**D**) *RP11-84C13.1* lncRNA masks *miR-576-3p* binding sites. ↑ and **↑**: increase; ↓: decrease; 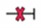: no repression; 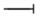: repression.

LncRNAs are very versatile regulators and they can play different roles in the nucleus and in the cytoplasm through various mechanisms. LncRNAs control the epigenetic state of genes and regulate both transcriptional (when they bind 3’UTR of mRNAs) and translation level (by base pairing at different sites in the coding and untranslated regions of mRNAs). Moreover, lncRNAs also have the ability to compete for miRNA-binding and to act as ‘sponges’ or ‘decoys’ for miRNAs, thus leading to enhanced translations of targeted transcripts [29,30]. Although the functional roles of miRNAs in osteoporosis have been extensively investigated [7,31,32], the role of lncRNAs in bone homeostasis and their underlying mechanism remains undefined [33]. Osteoporosis is not typically considered an immunological disorder albeit there are overlapping pathways between bone biology and biology of inflammation [34]. Emerging clinical and molecular studies suggest that inflammation exerts significant influence on bone turnover, inducing osteoporosis. Numerous pro-inflammatory cytokines have been implicated in the regulation of osteoblasts and osteoclasts, and a shift towards an activated immune profile has been hypothesized as an important risk factor [35]. In this paper, we report results of a pilot study to determine the expression profile of lncRNAs regulating pro-inflammatory and anti-inflammatory genes and miRNAs in osteoblast primary cells from OP patients vs. controls. Six lncRNAs were significantly down-regulated in OP patients and in silico bioinformatics analysis highlighted several predicted lncRNA targets (either miRNAs or mRNAs) implicated in bone homeostasis (Table 3 and Table 4). Our expression analysis showed the down-regulation in OP cells of *GAS5* lncRNA, whose role is well established in literature. *GAS5* lncRNA has been reported to be a key control element during growth, differentiation, and development in mammalian species. In a recent study, the role of *GAS5* in growth plate chondrocytes [36], bone marrow mesenchymal stem cells (BMSCs) [37], and human multipotential mesenchymal stem cells (hMSCs) [38] was demonstrated and this could suggest a role of *GAS5* in bone homeostasis. *GAS5* is predicted to negatively regulate *hsa-miR-21-5p* which also regulates osteoclast formation and differentiation through a positive feedback loop involving c-Fos/*miR-21*/PDCD4 (programed cell death 4) [31]. Furthermore, *miR-21-5p* was highly expressed in bone tissue obtained from osteoporosis patients [39,40]. One of putative targets of *hsa-miR-21-5p* is *PTX3* mRNA. *Pentraxin 3* (*PTX3*) gene encodes for the prototypic long pentraxin, which is released in response to primary pro-inflammatory stimuli and represents an essential player in tuning inflammation [41,42,43]. It has been proposed that PTX3 elevation during bone inflammatory conditions promotes RANKL production and favors osteoclastogenic potential by osteoblasts, implying its involvement in bone resorption [44]. In a recent study, Scimeca et al., demonstrated the down-regulation of PTX3 at both transcription and translation levels in OP osteoblast cell lines [45]. It is possible therefore to speculate that PTX3 decrease is a downstream effect, mediated by *miR-21-5p*, caused by the downregulation of *GAS5* lncRNA in osteoblasts from OP patients (Figure 3A) [29,30].

Moreover, bioinformatics analysis predicted the *CTC-487M23.5* lncRNA binding with the 3’UTR of *HDAC2* mRNA. HDAC2, the major enzymes for histone deacetylation, is a key positive regulator during receptor activator of nuclear factor-κB ligand (RANKL) and its overexpression leads to osteoclastogenesis and bone resorption. In particular, HDAC2 activates Akt thus suppressing FoxO1 transcription resulting in enhanced osteoclastogenesis [46]. Further functional studies are needed to demonstrate the *CTC-487M23.5* lncRNA/*HDAC2* mRNA interaction and its effect on the stability and translation of *HDAC2* mRNA (Figure 3B). 

Another interesting result is the interaction between *NCBP2-AS2* and several miRNAs deregulated in osteoporosis such as members of the *miR-497∼195* cluster (associated with the inhibition of osteoblastogenesis), *miR-103a* (regulating osteoblast differentiation), and *miR-23a/-27a/24-2* cluster (controlling osteogenesis) [28,39]. Thus, *NCBP2-AS2* lncRNA could indirectly regulate osteogenesis and bone formation through interaction with this set of miRNAs by targeting most of the known signaling pathways involved in bone homeostasis. For example, Wnt/β-catenin signaling is indispensable for osteoblastogenesis, and loss or gain of function of this pathway is associated with a profound decrease or increase of bone mass, FoxOs were found to be crucial for bone mass homeostasis even though they are not bone specific transcription factors, and the Hippo pathway is involved in regulating bone-forming osteoblasts during bone development and remodeling [47,48] (Figure 3C). Interestingly, bioinformatic analysis also reveals that *miR-103a* and *miR-23a/27a/24-2* clusters putatively interact with RUNX2, important regulatory factors involved in skeletal gene expression and osteoblastic differentiation [11]. Recent findings suggest that stabilized Runx2 induces Wnt signaling and enhances aerobic glycolysis in osteoblasts [49]. Moreover, Haxaire et al., [11] demonstrated that overexpression of Runx2 in experimental animals strongly induced RANKL, but depleted beta-catenin, reducing bone mass and bone volume. However, if the overexpression of Runx2 was performed in the presence of lithium chloride (an inhibitor of the beta-catenin degrading enzyme GSK-3 beta) osteoblasts could differentiate and bone volume was restored (described in a recent review by Kovács et al.) [50].

Finally, we found that *RP11-84C13.1* lncRNA interacts with *hsa-miR-576-3p*. This miRNA is implicated in the regulation of viral infection and inflammation [51]. Bioinformatic analysis reveals that one of *hsa-miR-576-3p* targets is *fibroblast growth factor 2* (*FGF2*) mRNA. FGF2 protein is expressed in the majority of cells and tissues including limb bud, chondrocyte and osteoblast cells, and it was experimentally demonstrated that deletion of *Fgf2* in mice leads to decreased bone mass, bone formation, and mineralization [52] (Figure 3D).

In conclusion, although the present findings require confirmation in larger samples, our study has documented for the first time the deregulation of six lncRNAs in primary osteoblasts from OP patients with a potential involvement in inflammation and bone homeostasis. Moreover, an in silico search for lncRNA targets uncovered several mRNAs and proteins representing novel potential biomarkers for this debilitating disease. Additional experiments on the functional and physiological role of these lncRNAs and their putative interactions are needed in order to understand the pathogenic mechanism of OP and to develop personalized therapeutic approaches for bone resorption.

## Figures and Tables

**Table 1:** Main characteristics of osteoporosis (OP) patients and control (CTR) individuals.

Clinical Characteristics	OP (*n* = 4)	CTR (*n* = 4)
BMI (kg/m^2^)	25.61 ± 1.13	27.0 ± 6.0
T Score (L1–L4)	−2.85 ± 0.15	0.95 ± 0.01
T Score (neck)	−2.77 ± 0.17	0.19 ± 0.05
PASE test	72.96 ± 24.89	100 ± 20.86
Kellgren–Lawrence scale	0–1	0

**Table 2:** Differentially-expressed long non-coding RNAs in OP patients *vs.* CTRs.

Long Non-Coding RNAs (LncRNAs)	Fold-Regulation	*p*-Value
***CEP83-AS1***	−8.75 ↓ down	0.014
***RP11-84C13.1***	−5.44 ↓ down	0.015
***CTC-487M23.5***	−5.39 ↓ down	0.004
***GAS5***	−4.86 ↓ down	0.006
***NCBP2-AS2***	−4.34 ↓ down	0.020
***SDCBP2-AS1***	−4.00 ↓ down	0.002

Fold-regulation represents fold-change results in a biologically meaningful way. Fold-change values greater than one indicate a positive- or an up-regulation, and the fold-regulation is equal to the fold-change. Fold-change values less than one indicate a negative- or down-regulation, and the fold-regulation is the negative inverse of the fold-change. The *p*-values were calculated based on a Student’s t-test of the three replicate 2^(−ΔCt)^ values for each gene in the control group and OP group [24]. ↓: down-regulation.

**Table 3:** List of interaction between lncRNAs and putative mRNAs.

List of mRNAs Interacting with lncRNAs					
LncRNA	mRNA	Ensemble ID	MinEnergy	Location	SumEnergy
***CEP83-AS1*** **(ENST00000623122)**	-	-	-	-	-
***CTC-487M23.5*** **(ENST00000602872)**	***AGO3***	ENST00000373191	−63.2	UTR3	−2550.9
	***RBM28***	ENST00000223073	−62.5	UTR3	−2038.0
	***PDK1***	ENST00000282077	−71.3	UTR3	−1994.5
	***PPM1L***	ENST00000498165	−72.6	UTR3	−1622.0
	***HDAC2***	ENST00000519065	−56.2	UTR3	−1573.2
***GAS5*** **(ENST00000450589)**	***SOD2***	ENST00000538183	−40.8	UTR3	−1798.0
	***FGFR1OP***	ENST00000366847	−38.2	UTR3	−1508.2
	***NOS1***	ENST00000317775	−47.2	UTR3	−1380.6
	***EIF4E***	ENST00000450253	−42.2	UTR3	−1298.0
	***LRRC27***	ENST00000392638	−42.9	UTR3	−1002.8
***RP11-84C13.1*** **(ENST00000603357)**	***IGFN1***	ENST00000335211	−39.1	CDS	−2316.5
	***RYR2***	ENST00000366574	−40.3	CDS	−1848.3
	***CCDC88A***	ENST00000436346	−35.2	UTR5	−1688.0
	***CACNG8***	ENST00000270458	−37.9	UTR3	−1488.5
	***RYR3***	ENST00000389232	−40.4	CDS	−1469.4
***NCBP2-AS2*** **(ENST00000602845)**	***SMAD2***	ENST00000402690	−34.5	UTR5	−1363.4
	***PTPN14***	ENST00000366956	−28.7	CDS	−1249.8
	***LRP1***	ENST00000243077	−31.0	CDS	−1132.5
	***PTPN4***	ENST00000263708	−28.5	CDS	−1056.9
	***IGSF10***	ENST00000282466	−29.0	CDS	−1056.2
***SDCBP2-AS1*** **(ENST00000446423)**	***ASXL2***	ENST00000435504	−38.9	CDS	−4456.0
	***CBL***	ENST00000264033	−37.5	UTR3	−3988.8
	***HIVEP3***	ENST00000372584	−38.1	CDS	−3670.8
	***HDAC9***	ENST00000406451	−38.9	CDS	−3554.0
	***ROCK1***	ENST00000399799	−39.0	UTR5	−3065.6

(-): no mRNA interaction found.

**Table 4:** List of interactions between lncRNAs and putative miRNA-targeted proteins and pathways.

List of miRNAs Interacting with lncRNA				
LncRNA	miRNA	Pr.Score*	KEGG Pathways	Gene Target
***CEP83-AS1*** **(ENST00000623122)**	-	-	-	-
***CTC-487M23.5*** **(ENST00000602872)**	***hsa-miR-136-3p***	0.451	**ECM-receptor interaction**	***THBS2***
				***PPP1CB***
				***PDGFC***
***GAS5*** **(ENST00000450589)**	***hsa-miR-21-5p***	0.382	**Hippo signaling pathway FoxO signaling pathway**	***PDCD4***
				***PTX3***
***RP11-84C13.1*** **(ENST00000603357)**	***hsa-miR-576-3p***	0.408	**Hippo signaling pathway FoxO signaling pathway**	***FGF2***
				***FRS2***
				***PTPN11***
***NCBP2-AS2*** **(ENST00000602845)**	***hsa-miR-103a-3p***	0.734	**Hippo signaling pathway FoxO signaling pathway Wnt signaling pathway TGF-β signaling pathway mTOR signaling pathway HIF-1 signaling pathway AMPK signaling pathway Insulin signaling pathway TNF signaling pathway**	***Runx2***
	***hsa-miR-497-5p***	0.799		***BMP***
	***hsa-miR-195-5p***	0.662		
	***hsa-miR-23a-3p***	0.417		***Runx2/SATB2***
	***hsa-miR-24-2-5p***	0.497		
	***hsa-miR-23b-3p***	0.418		***SMAD***
	***hsa-let-7d-3p***	0.391		***Collagens***
***SDCBP2-AS1*** **(ENST00000446423)**	***hsa-miR-2116-3p***	0.545	**FoxO signaling pathway TGF-β signaling pathway**	
	***hsa-miR-532-3p***	0.558		
	***hsa-miR-150-5p***	0.644		

* Pr. Score (or miTG score) is a general score for the predicted interaction, the closer to 1, the greater the confidence. The higher miTG score corresponds to a high probability of targeting [21] (-): no miRNA and gene interaction found.

## Abbreviations

OP	Osteoporosis
CTR	Control
lncRNAs	Long non-coding RNAs
miRNAs	Micro-RNAs
PASE	Physical Activity Scale for the Elderly
Pr. Score	Prediction Score
